# Research on spinal characteristics and exercise intervention in 6–18 year old adolescents based on computer vision recognition

**DOI:** 10.3389/fpubh.2025.1641479

**Published:** 2026-01-15

**Authors:** Xiangrong Cheng, Jingmin Liu, Yu Liu, Yukihiro Haswgawa, Jianyu Li, Mingliang Ye, Fei Xiao

**Affiliations:** 1 Department of Physical Education, Qingdao University of Technology, Qingdao, Shandong, China; 2 Department of Physical Education, Tsinghua University, Beijing, China

**Keywords:** computer vision, China, adolescents, spine, intervention

## Abstract

**Introduction:**

Spinal health significantly impacts adolescents’ posture, athletic performance, mental well-being, and quality of life. It provides data support for promoting the physical and mental health of adolescents and implementing the construction of a strong education country.

**Methods:**

This study employed computer vision recognition technology to screen and evaluate the spinal health status of 4,534 adolescents aged 6–18. Through a 12-week exercise intervention, the study compared the effects of different exercise programs on adolescents’ spinal health.

**Results:**

(1) The spine characteristics of 4,534 children and adolescents aged 6 ~ 18 years old showed that there were more middle and high risk people with neck forward tilt, neck roll, overall spine roll, high and low shoulder and pelvic rotation, and the tilt or rotation angle of boys was larger than that of girls. (2) From the perspective of age, the tilt angle of children and adolescents in the low age group and 18 years old is larger. (3) Football, badminton and dance can improve the spine tilt angle of adolescents, but different sports have different effects on different parts of the spine.

**Conclusion:**

(1) Children and adolescents aged 6–18 have different degrees of problems in the neck, chest, waist, spine, shoulder joint and pelvis. (2) There are age and gender differences in spine health of adolescents aged 6–18 years old, and the problems of low age group and 18 years old are more prominent. (3) When using exercise to improve spinal health issues, it is essential to select different types of sports based on the tilt of different body parts and arrange the exercise intensity reasonably according to individual health conditions and physical capabilities.

## 1 Introduction

The spine is a crucial supportive structure of the human body, not only bearing the body’s weight but also protecting the spinal cord, a vital component of the central nervous system. The physiological curvature of the spine is straight from the front, but from the side, it is composed of multiple bends, forming an S-shaped or C-shaped curve (1). Specifically, the cervical spine has a forward lordosis, the thoracic spine has a backward kyphosis, and the lumbar spine has a forward lordosis. This physiological curvature helps disperse body weight and absorb shocks, reducing the pressure on the spinal cord and nerves. Adolescence represents a critical period for spinal growth and development, with spinal health directly influencing adolescents’ posture, athletic ability, mental well-being, and future quality of life (2, 3). Spinal tilt is considered to be a spinal health problem because it may cause a series of physiological and functional abnormalities, which in turn leads to instability of the spinal structure and related diseases. Maintaining spinal health during this stage helps prevent spinal diseases in adulthood and enhances adolescents’ self-confidence and mental well-being. One reason for the neglect of adolescent spinal health is the choice of testing methods, as existing testing equipment often suffers from high costs, radiation exposure, complex operation, and high demands on the tester’s professional expertise. All kinds of sports have the effect of improving bad spinal problems, but the degree of improvement is not clear. This study utilized a computer vision recognition technology-developed adolescent spinal health tester to measure the current spinal health status of adolescents. This approach not only allows for a broad understanding of spinal health levels across different age groups but also reduces the operational requirements for testers and the level of cooperation needed from adolescents. By assessing the spinal health levels of adolescents, this study aims to identify the main issues currently affecting adolescent spines and employ reasonable exercise intervention methods to improve spinal health, laying the foundation for addressing poor spinal health in adolescents and providing data support for the implementation of a strong education initiative.

## 2 Research objects and methods

### 2.1 Research objects

This study used a multi-stage stratified cluster random sampling method to test the spine status of 4,534 children and adolescents aged 6–18 years in 9 schools. The tested children and adolescents were all students aged 6 to 18 from urban primary schools, middle schools, high schools, and first-year university students in Qingdao. Four classes were randomly selected from each grade level in every school. All subjects had no previous surgical history of spine-related diseases, and participated voluntarily with the consent of their guardians. They were approved by the Medical Committee of the Science and Technology Ethics Committee of Tsinghua University (THU01-20240130).

### 2.2 Research methods

#### 2.2.1 Testing method

A human spine assessment device was employed to measure and evaluate the body posture of the survey participants. The instrument used in this study is a human spine tester developed by Holomotion, which has been verified by professionals. The accuracy of this testing instrument has been compared against a professional motion capture system as the gold standard. The Holomotion accuracy evaluation report indicates that its predicted values show a high degree of consistency with the angular change trends of the gold standard, demonstrating high reliability. Its accuracy level meets the requirements for clinical assessments in various scenarios. The subjects wore tight-fitting clothing and stood naturally in front of the screen, with measurements taken from three directions: front, left side, and right side. The assessment device, based on human functional anatomical standards and spatial data coordinates collected by a depth camera, extracted body landmark data from the human images through intelligent background computation. It then conducted a segmented evaluation of the human trunk and generated a visual data report. The assessment indicators mainly included the deviation magnitude, deviation angle, deviation direction, and risk levels of different spinal segments, as well as the shoulders and pelvis.

The testing process is divided into the following seven steps:

Step 1: Introduce the purpose, process, and precautions of the test to the subject.
Step 2: Measure the height and weight of the subject.
Step 3: The tester enters the height, weight, gender, and age of the subject into the body posture assessment device.
Step 4: The subject stands with their feet together, hands naturally placed on either side of their body, eyes looking straight ahead, and first faces the testing instrument for 10 s.
Step 5: Stand with your left side facing the testing device for 10 s.
Step 6: Stand with the right side of your body facing the testing instrument for 10 s.
Step 7: Save the data after completing the three-direction test.

#### 2.2.2 Experimental method

A total of 169 eighteen-year-old adolescents were randomly selected as intervention subjects, and the subjects chose the football group, badminton group, or dance group according to their preferences. Before the intervention, there were no significant differences in the degree of spinal abnormalities among the three groups of subjects. Before the exercise intervention, all subjects were measured for their spinal curvature status. After the test was completed, a 12-week exercise intervention was conducted three times a week. In addition to warm-up and relaxation, exercise intervention for 1 h per class. After 12 weeks, all subjects were measured again for their spinal curvature status. During the 12-week exercise intervention, the football group only engaged in football exercises, mainly focusing on passing and catching, shooting, and playing matches; the badminton group only practices badminton, mainly focusing on serving, receiving, hitting high and far balls, and competing; the Dance team Just dance to the music.

#### 2.2.3 Mathematical statistics method

The collected data were organized using the statistical software SPSS27, and statistical analysis was conducted using methods such as single-sample *t*-tests, and paired-sample *t*-tests. A paired sample *t*-test was used to compare the differences in spinal segment inclination angles between male and female students. A single sample *t*-test was employed to compare the differences before and after interventions involving different sports activities.

## 3 Results

### 3.1 Demographic characteristics of participants

The demographic data of all subjects are shown in Tables 1, 2. A total of 4,534 children and adolescents aged 6–18 years participated in the test. The average height was 160.10 ± 16.17 cm, the average weight was 53.46 ± 18.78 kg, and the average BMI was 20.25 ± 5.25.

**Table 1:** Training schedule for different intervention groups.

Group	Training period	Training frequency	Session duration	Training content
Football team	12 weeks	3 times/week	1 h	First month: Passing, receiving Second month: dribbling, shooting Third month: matches Fourth month: matches
Badminton team	12 weeks	3 times/week	1 h	First month: overhead clears Second month: Serving, receiving serves Third month: matches Fourth month: matches
Dance team	12 weeks	3 times/week	1 h	First month: Complete dance routines-1 Second month: Complete dance routines-2 Third month: Complete dance routines-1 Fourth month: Complete dance routines-1

**Table 2:** Demographic data of all participants ( $\bar{X}\pm \mathrm{SD}$ ).

Age	Number of participants (n)	Number of boys (n)	Number of girls (n)	Height (cm)	Weight (kg)	BMI
6	192	96	96	126.11 ± 7.96	25.63 ± 5.27	16.00 ± 2.19
7	206	116	90	131.18 ± 7.22	28.56 ± 6.98	16.40 ± 2.68
8	300	166	134	136.78 ± 7.65	31.39 ± 8.37	16.64 ± 3.39
9	226	102	124	140.78 ± 9.49	35.99 ± 11.15	17.86 ± 3.90
10	270	140	130	150.38 ± 8.58	41.73 ± 11.74	18.24 ± 3.84
11	428	242	186	155.07 ± 10.56	45.53 ± 12.46	19.15 ± 9.15
12	348	190	158	161.98 ± 8.11	53.66 ± 13.81	20.28 ± 4.14
13	372	188	184	166.27 ± 7.14	58.36 ± 15.20	20.94 ± 4.47
14	348	174	174	169.89 ± 8.08	61.90 ± 14.55	21.22 ± 4.48
15	404	118	286	169.60 ± 7.18	64.77 ± 16.55	22.32 ± 5.43
16	452	190	262	172.00 ± 9.09	66.55 ± 17.26	22.38 ± 4.99
17	308	104	204	171.66 ± 8.43	64.56 ± 14.67	21.79 ± 4.12
18	680	288	392	170.19 ± 8.18	63.36 ± 12.57	21.76 ± 3.29
Total	4,534	2,114	2,420	160.10 ± 16.17	53.46 ± 18.78	20.25 ± 5.25

### 3.2 Spinal characteristics of adolescents aged 6–18 years

#### 3.2.1 Neck characteristics of adolescents aged 6–18

**Figure 1:**
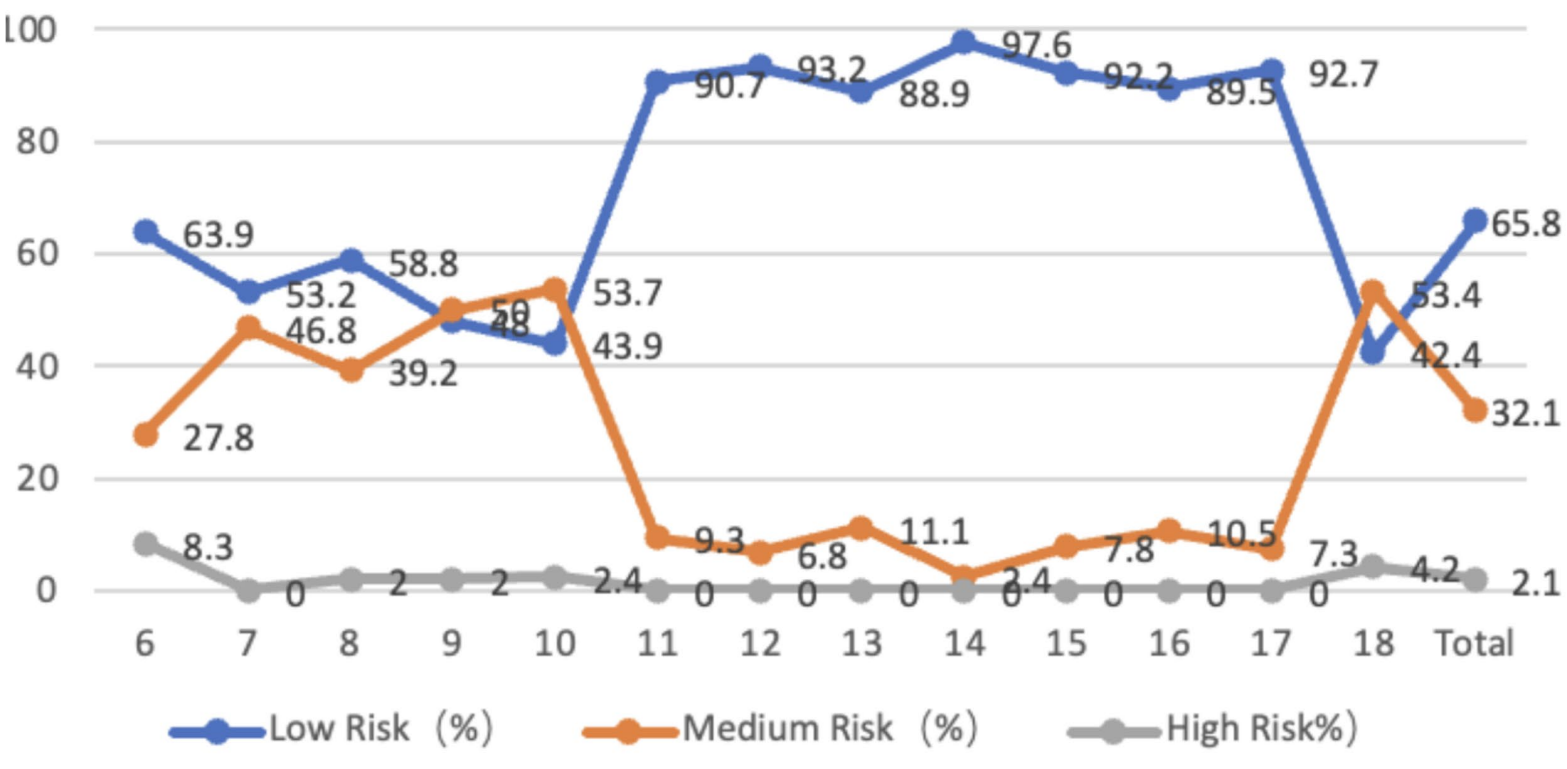
Line graph of the proportion of adolescents at risk for sagittal plane neck abnormalities by age.

The sagittal plane of the neck of adolescents aged 6–18 years is entirely forward-tilted, with an average inclination angle of 8.98 degrees. Among them, 32.1% of adolescents are at moderate risk, and 2.1% are at high risk. In terms of age, the problems of teenagers in primary school and 18-year-olds are more serious. From the perspective of gender differences, there is no significant difference in the forward tilt of the neck between boys and girls in general (*p* > 0.05). The average forward tilt angle of the neck for boys is 9.08 degrees, while that for girls is 8.89 degrees. The forward tilt angle for boys is larger than that for girls, but there is a significant difference in the forward tilt of the neck between boys and girls at the ages of 6, 11, 14, and 18 (*p* < 0.05) (Figure 1).

**Figure 2:**
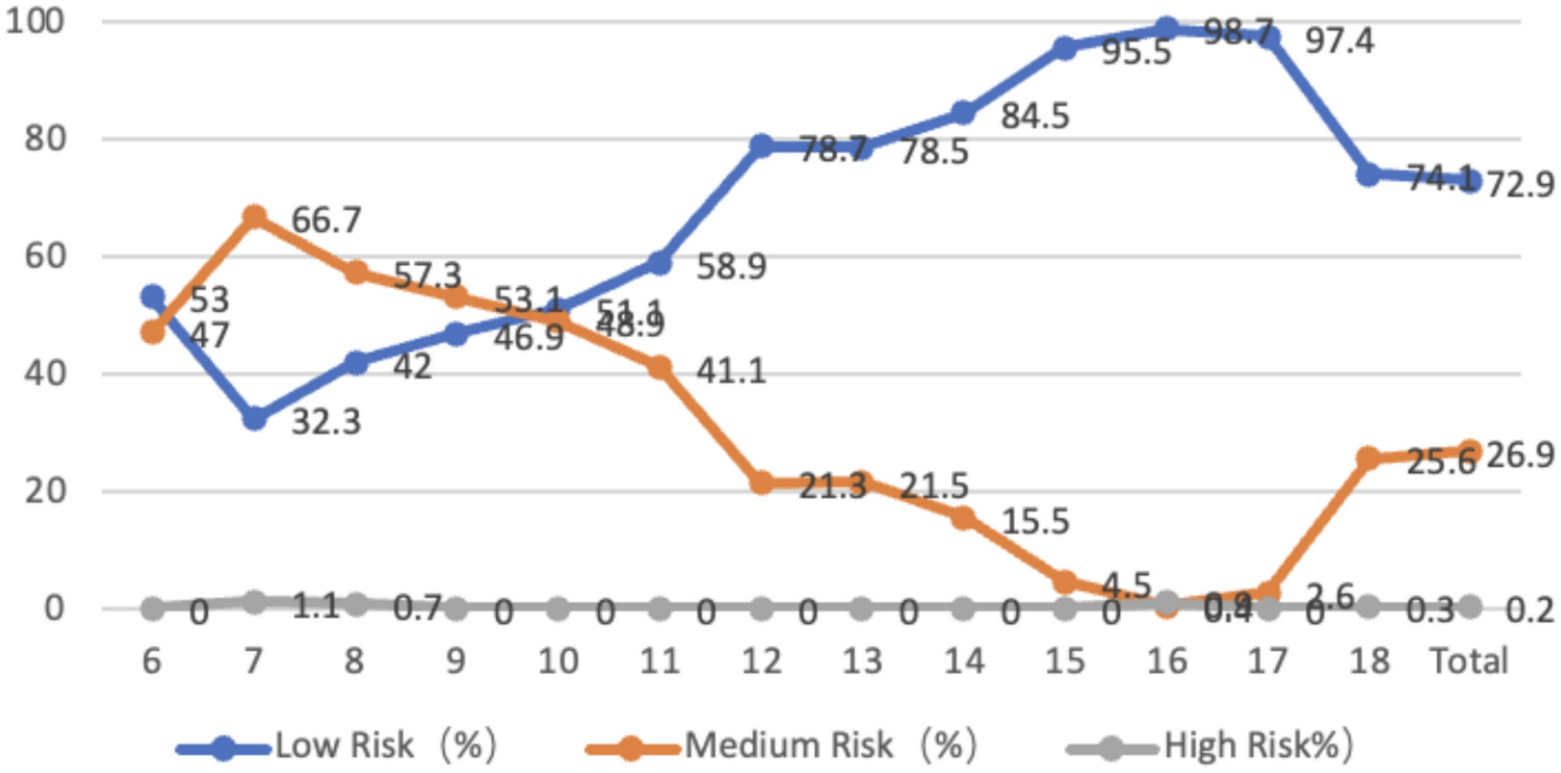
Line graph of the proportion of adolescents at risk for coronal plane neck abnormalities by age.

95.5% of the adolescents aged 6–18 years old had a rightward tilt in the coronal plane of the neck, while only 4.5% had a leftward tilt. The average leftward tilt angle was 1.66 degrees, while the average rightward tilt angle was 3.47 degrees. The rightward tilt angle was greater than the leftward tilt angle, with an average neck score of 47.67 points. 26.9% of the adolescents were at moderate risk, while 0.2% were at high risk. In terms of age, adolescents in primary school and those aged 18 have higher severity levels. From the perspective of gender differences, the average leftward tilt angle for male students is 0.85 degrees, while the average rightward tilt angle for female students is 2.03 degrees. There is no significant difference between male and female students (*p* > 0.05); The average rightward tilt angle for boys is 3.82 degrees, while for girls it is 3.16 degrees. The rightward tilt angle for boys is significantly higher than that for girls (*p* < 0.01). In addition, except for the age of 6, the rightward tilt angle of boys’ necks is greater than that of girls’ necks in all age groups, with significant differences observed at the ages of 10, 14, and 17 (*p* < 0.05) (Figure 2).

The average angle between the cervical vertebrae in the coronal plane for adolescents aged 6–18 is 178.16 degrees, with an average score of 21.41. The proportion of individuals in the low-risk state is 96.7%, while the proportion in the medium-risk state is 3.3%. In terms of age, there are more people with medium risk at the age of 6, 7, and 15. From the perspective of gender, the average inclination angle of the cervical angle in the coronal plane is 178.10 degrees for boys and 178.22 degrees for girls, with no significant difference between boys and girls (*p* > 0.05). There is a significant difference in the inclination angle between boys and girls at the age of 13 and 15, with girls having a greater inclination angle than boys.

#### 3.2.2 Chest characteristics of adolescents aged 6–18

**Figure 3:**
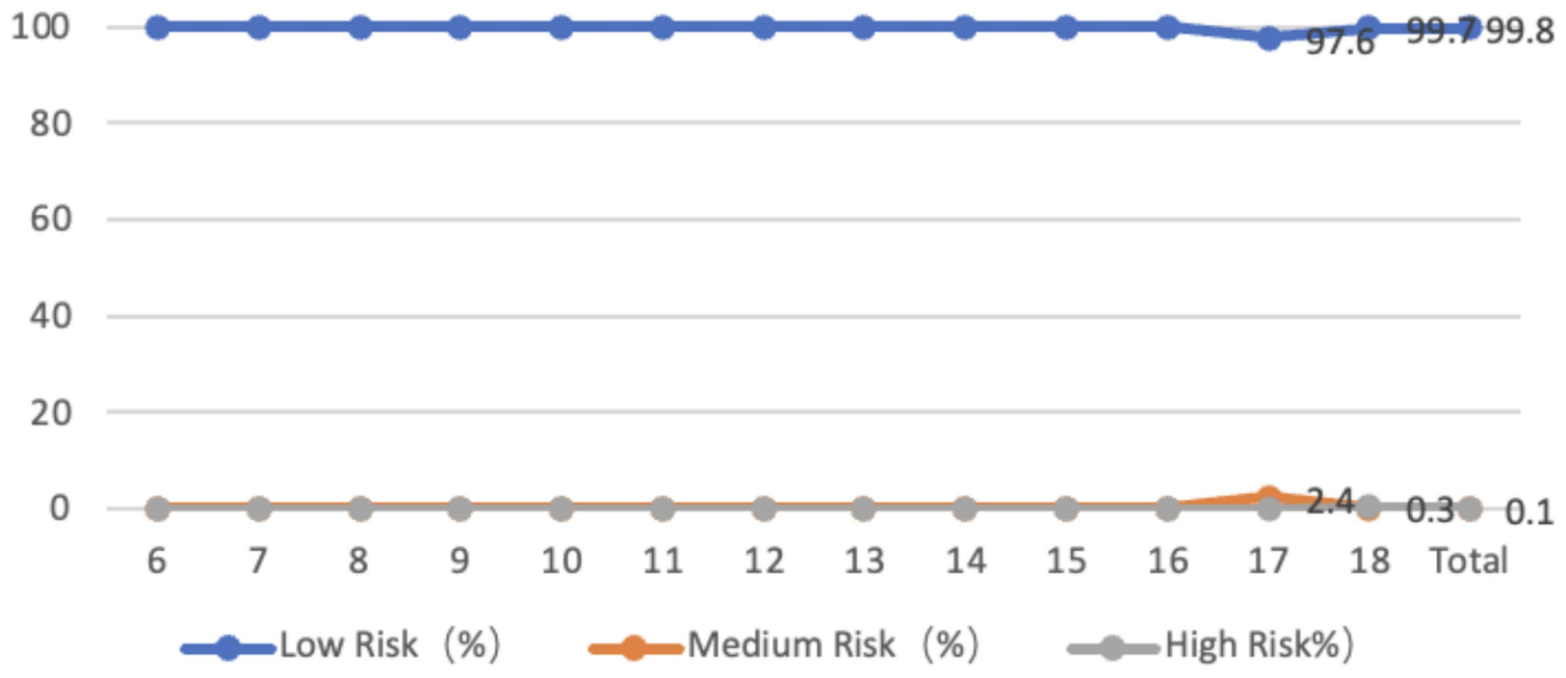
Line graph of the proportion of adolescents at risk for sagittal plane thoracic abnormalities by age.

The sagittal plane characteristics of the chest in adolescents aged 6–18 years showed that 68.6% of the adolescents had a forward tilt of the chest, with an average tilt angle of 2.12 degrees, 31.4% had a backward tilt of the chest, with an average tilt angle of 1.77 degrees, and 0.2% were at a medium-high risk state, all of whom were 17 and 18 years old. From the perspective of gender differences, the average forward inclination angle of boys is 2.27 degrees, while that of girls is 1.95 degrees. There is a significant difference in the forward inclination of the chest between boys and girls (*p* < 0.01), and the inclination angle of boys is greater than that of girls. There is no significant difference in the backward inclination angle between boys and girls (Figure 3).

**Figure 4:**
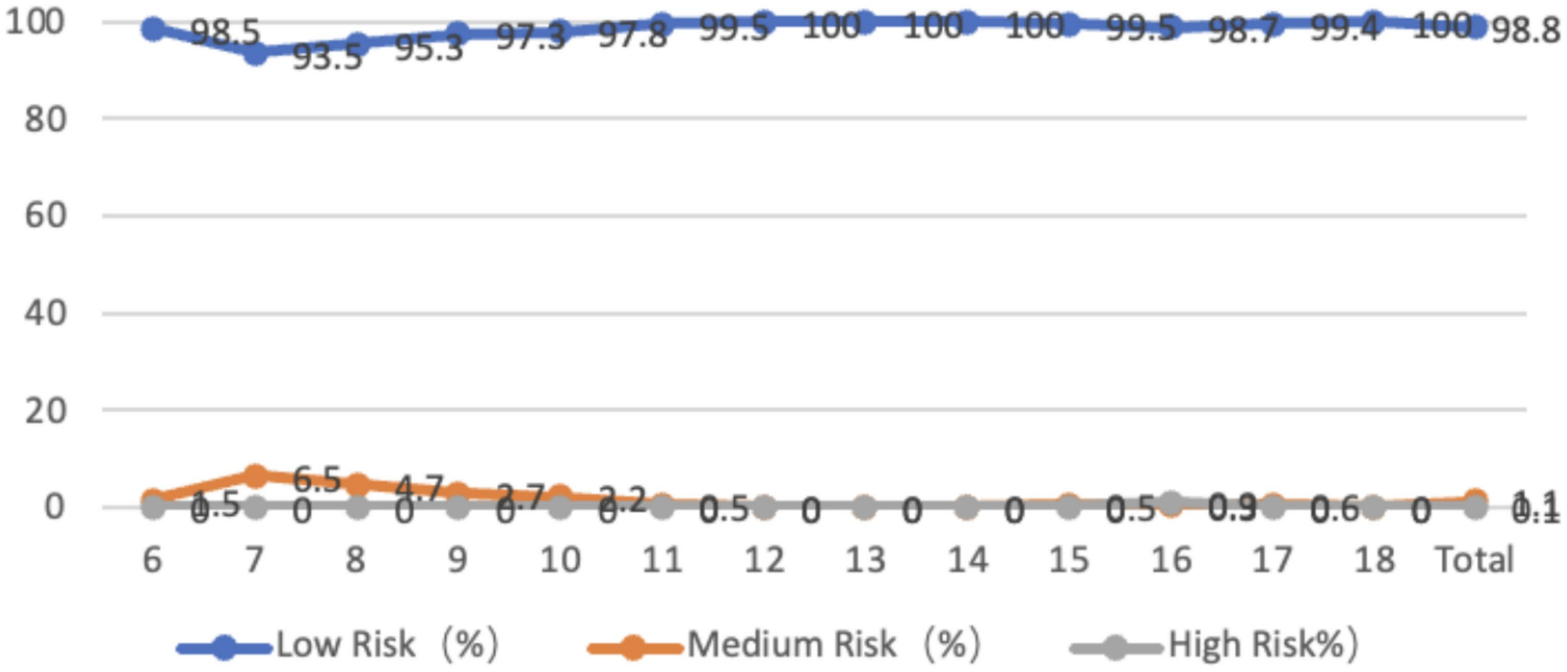
Line graph of the proportion of adolescents at risk for coronal plane thoracic abnormalities by age.

The chest coronal plane characteristics of adolescents aged 6–18 showed that 40.8% of the adolescents had a leftward tilt of the chest, with an average tilt angle of 40.80 degrees, 59.2% of the adolescents had a rightward tilt of the chest, with an average tilt angle of 1.47 degrees, and 1.2% of the adolescents were at a medium-high risk state, mostly in the lower age group. Adolescents in the higher age group tend to have a more leftward tilt of their chests, while those in the lower age group tend to have a more rightward tilt of their chests, and the chest scores of the lower age group are generally higher. From the perspective of gender, there is a significant difference between boys and girls in the leftward inclination of their chests (*p* < 0.01). The average inclination angle for boys is 0.96 degrees, while that for girls is 1.37 degrees, with girls having a greater inclination angle than boys. There is also a significant difference between boys and girls aged 17 and 18; There is no significant difference in the rightward tilt of the chest between boys and girls (Figure 4).

The average inclination angle of the thoracic angle in the coronal plane for adolescents aged 6 to 18 years old is 176.54 degrees, with 71.9% in the low-risk state, 28% in the medium-risk state, and 0.1% in the high-risk state. The condition is more severe for those aged 6 to 10 years old. From the perspective of gender, the average inclination angle of boys is 176.42 degrees, while that of girls is 176.65 degrees, with a significant difference between boys and girls (*p* < 0.01). There are significant differences between boys and girls at the age of 9, 10, and 11, and girls are larger than boys.

#### 3.2.3 Lumbar characteristics of adolescents aged 6–18

**Figure 5:**
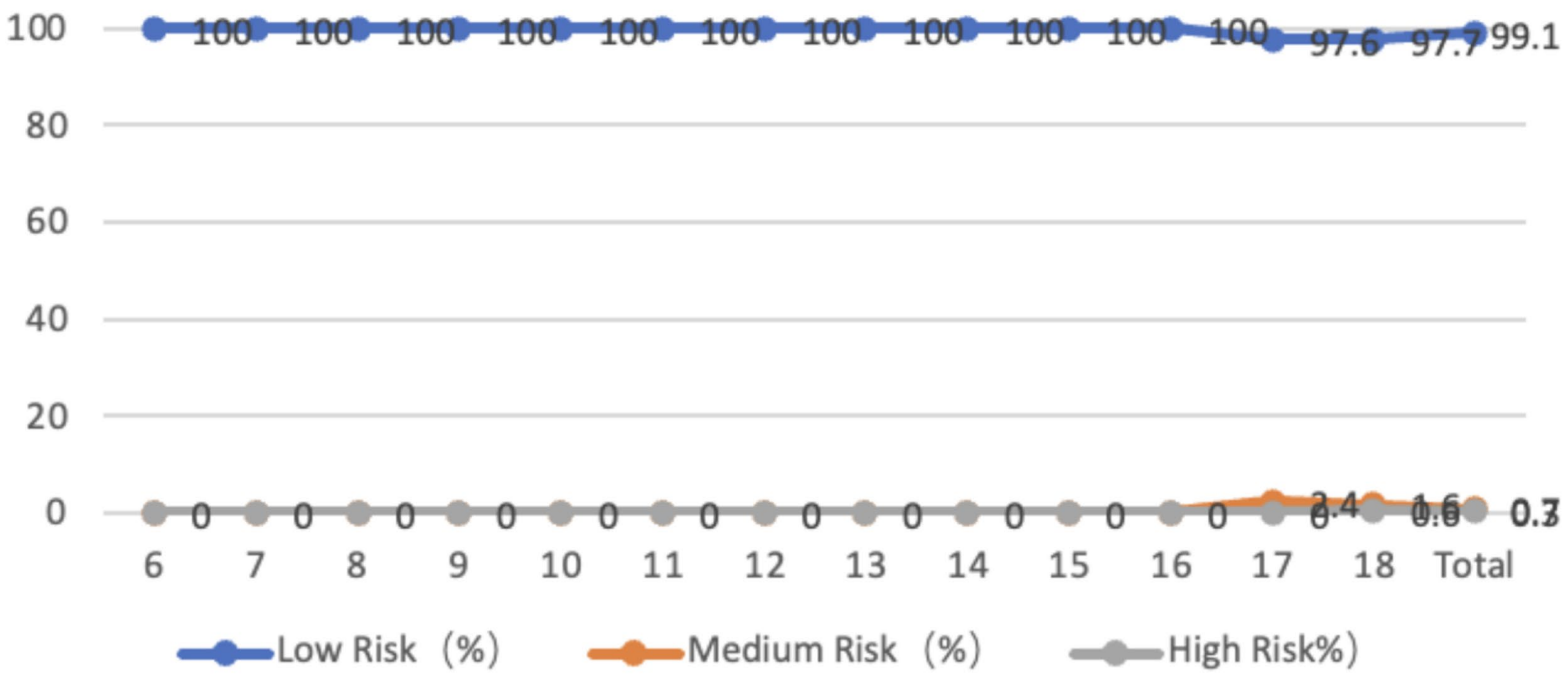
Line graph showing the proportion of adolescents at risk for lumbar sagittal plane abnormalities by age.

The sagittal plane characteristics of the waist of adolescents aged 6–18 showed that 98.9% of them had a backward tilt of the waist, with an average tilt angle of 4.65 degrees. The tilt angle was smaller in the middle age group, with the largest tilt angle at 18 years old. 1% of the adolescents were at medium-high risk, and all were 17- and 18-year-olds. From the perspective of gender, there is a significant difference in the backward inclination of the waist between boys and girls (*p* < 0.01), and the inclination angle of girls is greater than that of boys. The maximum backward inclination angle of the waist is observed in boys and girls aged 18 years old (Figure 5).

**Figure 6:**
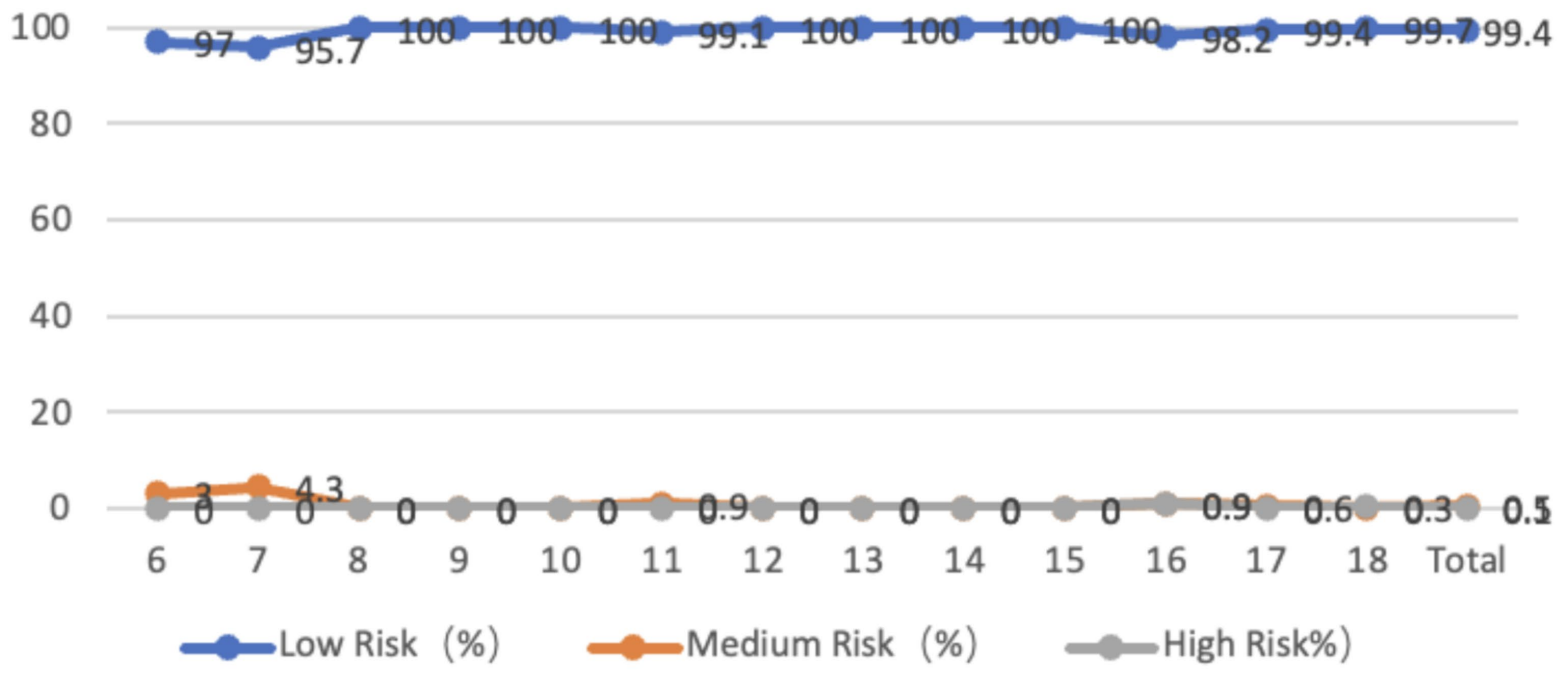
Line graph showing the proportion of adolescents at risk for lumbar coronal plane abnormalities by age.

The characteristics of the lumbar coronal plane in adolescents aged 6–18 show that 36.8% of adolescents tilt to the left, with an average tilt angle of 1.26 degrees, and 63.2% of children and adolescents tilt to the right, with an average tilt angle of 1.33 degrees. Overall, the tilt angle decreases with increasing age, and 0.6% of children and adolescents are at a medium-high risk state. From the perspective of gender, there is no significant difference between boys and girls, whether leaning left or right (Figure 6).

The average inclination angle of the lumbar angle in the coronal plane for adolescents aged 6 to 18 years old is 178.58 degrees, with a medium-high risk proportion of 0.9%, and both are in the low-age group and 18 years old. From the perspective of gender, the average inclination angle of boys is 178.68 degrees, while that of girls is 178.49 degrees, with a significant difference between boys and girls (*p* < 0.01). There are significant differences between boys and girls aged 15, 17, and 18 years old.

#### 3.2.4 Overall characteristics of the spine in adolescents aged 6–18 years

**Figure 7:**
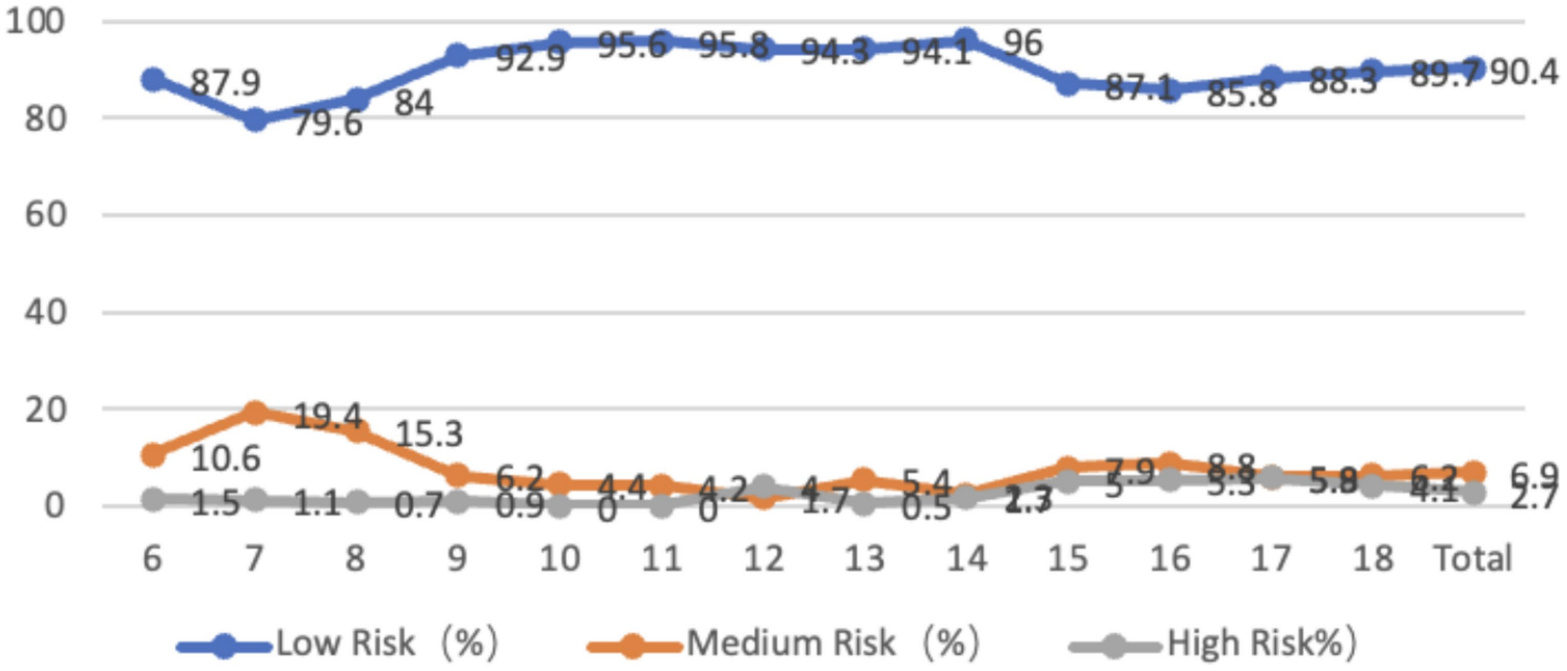
Line graph of the proportion of adolescents at risk for overall spinal coronal plane abnormalities by age.

The overall leftward inclination of the spine in adolescents aged 6–18 years old accounted for 20.1%, with an average inclination angle of 4.88 degrees. The rightward inclination accounted for 79.9%, with an average inclination angle of 2.95 degrees, and the overall spine score was 39.85 degrees. Among them, the low-risk state accounted for 90.4%, the medium-risk state accounted for 6.9%, and the high-risk state accounted for 2.7%. In terms of age, the proportion of high-risk cases is higher in the low and high age groups, while the proportion of high-risk cases is relatively low in the 10–14 age group, with a lower inclination angle. From the perspective of gender, the average leftward inclination angle of the spine in the coronal plane is 4.66 degrees for male students and 5.00 degrees for female students, with no significant difference between the two groups (*p* > 0.05); The average rightward tilt angle for males is 2.70 degrees, while for females it is 3.20 degrees, with a significant difference between males and females (*p* < 0.05) (Figure 7).

#### 3.2.5 Characteristics of the shoulder joint in adolescents aged 6–18

**Figure 8:**
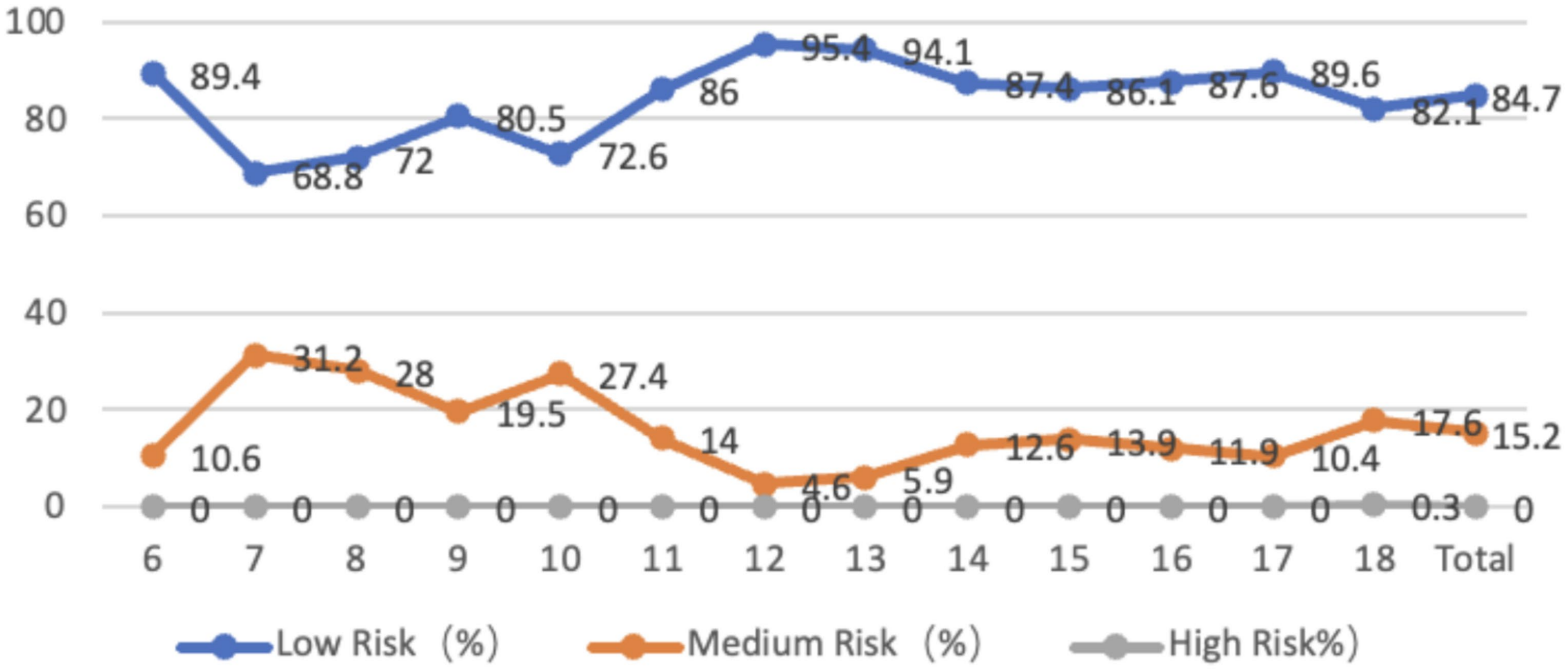
Line graph of the proportion of adolescents at risk for shoulder coronal plane abnormalities by age.

The shoulder characteristics of adolescents aged 6–18 show that only 0.1% of the adolescents have no shoulder tilt, 96% of the adolescents have a left shoulder tilt, with an average tilt angle of 1.76 degrees, 3.9% of the adolescents have a right shoulder tilt, with an average tilt angle of 0.68 degrees, and 15.3% of the adolescents are at medium-high risk. The proportion of adolescents at medium-high risk is higher among younger and 18-year-old adolescents, and the tilt angle is larger. From the perspective of gender, the average inclination angle of boys with left shoulder height is 1.90 degrees, while that of girls is 1.64 degrees. There is a significant difference between boys and girls (*p* < 0.01), and boys have a larger inclination angle than girls. There is no significant difference between boys and girls with right shoulder height (*p* > 0.05) (Figure 8).

#### 3.2.6 Pelvic characteristics of adolescents aged 6–18 years

**Figure 9:**
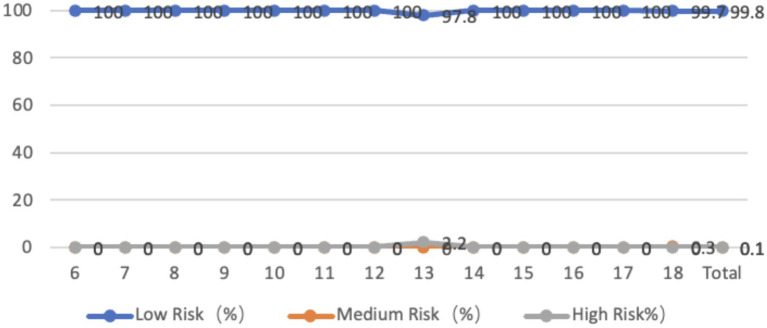
Line graph showing the proportion of adolescents at risk for pelvic sagittal plane abnormalities by age.

The number of adolescents aged 6–18 years with a forward tilt in the sagittal plane of the pelvis is 100%, with an average tilt angle of 5.64 degrees. The tilt angle is highest in 18-year-olds, and 0.2% of adolescents are at a medium-high risk. From the perspective of gender, the average forward inclination angle of the pelvis is 5.29 degrees for boys and 5.93 degrees for girls. There is a significant difference in the forward inclination angle of the pelvis between boys and girls (*p* < 0.05), and girls are significantly higher than boys. Both boys and girls have the largest inclination angle at the age of 18 (Figure 9).

**Figure 10:**
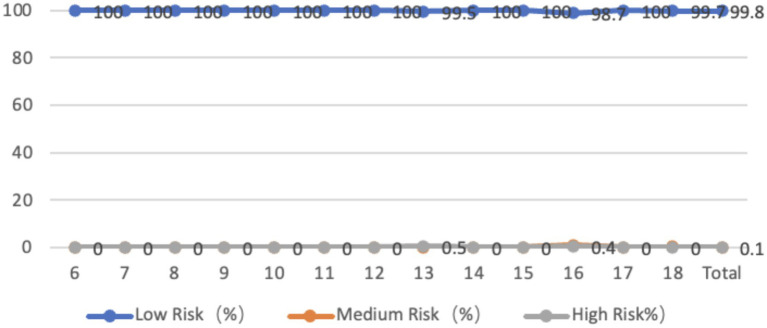
Line graph showing the proportion of adolescents at risk for pelvic coronal plane abnormalities by age.

Among adolescents aged 6–18, only 0.3% have a normal pelvis, while 98.9% have a leftward tilt, with an average tilt angle of 1.95 degrees. 0.8% have a rightward tilt, with an average tilt angle of 1.05 degrees. 99.8% of adolescents’ pelvic coronal planes are in a low-risk state, while 0.2% are in a medium-high risk state. In terms of age, the average inclination angle is larger for younger (6–10 years old) and 18-year-olds. From the perspective of gender, the average leftward inclination angle of the pelvis for male students is 1.95 degrees, while that for female students is 1.95 degrees. The average rightward inclination angle of the pelvis for male students is 1.38 degrees, while that for female students is 0.75 degrees. There is no significant difference between male and female students (*p* > 0.05) (Figure 10).

**Figure 11:**
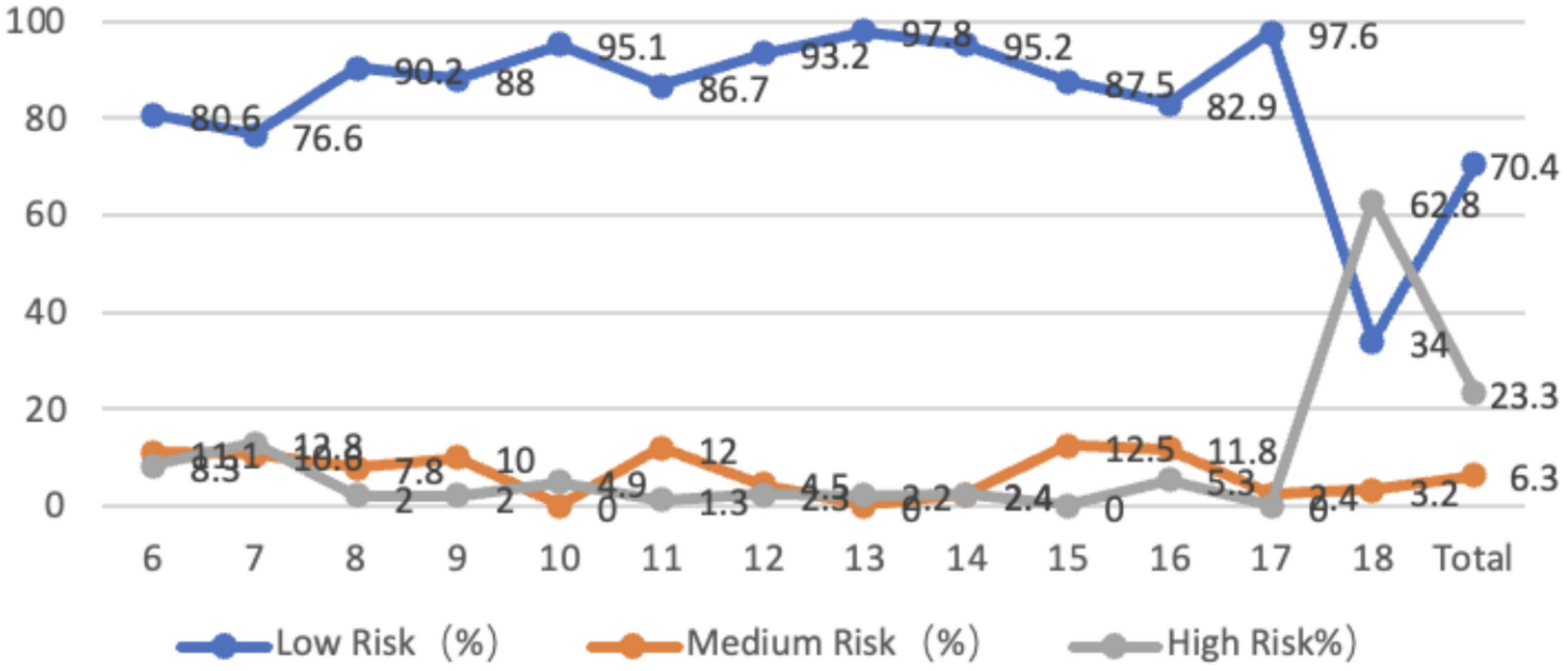
Line graph showing the proportion of adolescents at risk for pelvic horizontal plane abnormalities by age.

The survey of the characteristics of the pelvis horizontal plane in adolescents aged 6 to 18 showed that 25.6% of the adolescents had left rotation in the horizontal plane, with an average angle of 7.53 degrees. 74.4% of the adolescents had right rotation, with an average inclination angle of 10.28 degrees. 70.4% of the adolescents had low-risk pelvic horizontal plane inclination, 6.3% were at medium risk, and 23.3% were at high risk. The rotation angle was larger in younger age groups and at age 18. From the perspective of gender, the average left-handed angle for boys is 8.36 degrees, while the average left-handed angle for girls is 6.85 degrees. The average right-handed angle for boys is 11.00 degrees, while the average right-handed angle for girls is 9.65 degrees. There is no significant difference between boys and girls in terms of pelvic left-handedness or right-handedness, *p* > 0.05, but the inclination angle for boys is greater than that for girls, and the right-handed angle for 18-year-old boys is significantly greater than that for girls (*p* < 0.01) (Figure 11).

### 3.3 Exercise intervention promotes adolescent spinal health

As shown in Tables 3–5, football, badminton, and aerobics all significantly improved the forward tilt angle of the neck and the right tilt angle of the neck (*p* < 0.05). All three sports also improved the right tilt angle of the chest, with football and badminton significantly reducing it (*p* < 0.01). All three sports also significantly improved the backward tilt angle of the waist (p < 0.01), as well as the right tilt angle of the waist. However, the forward tilt angle of the chest sagittal plane increased in all three sports, with significant differences between football and badminton (*p* < 0.01).

**Table 3:** Comparison of neck tilt angle before and after intervention in each group.

Group	Neck sagittal plane Forward tilt angle			Neck coronal plane Rightward tilt angle		
	Before intervention	After intervention	*P*	Before intervention	After intervention	*P*
Football team	11.19 ± 4.48	6.16 ± 2.30	0.001**	3.89 ± 2.25	3.30 ± 1.76	0.005**
Badminton team	12.92 ± 4.65	6.17 ± 1.82	0.001**	3.91 ± 1.69	2.42 ± 1.53	0.028*
Dance team	9.94 ± 3.76	5.95 ± 2.04	<0.001**	3.15 ± 1.58	2.53 ± 2.71	0.036*

Annotation: **P < 0.01, *P < 0.05.

**Table 4:** Comparison of chest tilt angle before and after intervention in each group.

Group	Chest sagittal plane Forward tilt angle			Chest coronal plane Rightward tilt angle		
	Before intervention	After intervention	*P*	Before intervention	After intervention	*P*
Football team	2.19 ± 1.36	2.87 ± 1.13	0.005**	0.99 ± 0.73	0.42 ± 0.25	<0.001**
Badminton team	1.51 ± 0.61	2.73 ± 1.09	0.009**	1.16 ± 0.88	0.26 ± 0.22	<0.001**
Dance team	1.82 ± 2.52	2.33 ± 1.26	0.454	0.83 ± 0.66	0.77 ± 2.15	0.531

**P < 0.01.

**Table 5:** Comparison of waist tilt angle before and after intervention in each group.

Group	Waist sagittal plane Backward tilt angle			Waist coronal plane Rightward tilt angle		
	Before intervention	After intervention	*P*	Before intervention	After intervention	*P*
Football team	6.60 ± 1.28	3.15 ± 0.71	<0.001**	0.69 ± 0.59	0.44 ± 0.49	0.183
Badminton team	6.50 ± 1.18	3.73 ± 0.97	<0.001**	1.02 ± 0.61	0.89 ± 0.33	0.458
Dance team	7.16 ± 3.11	3.35 ± 0.74	<0.001**	0.83 ± 0.69	0.59 ± 0.42	0.133

**P < 0.01.

From Table 6, it can be seen that all three types of exercise have improved the left shoulder height, but the right shoulder height has increased, with football and aerobics being more significant (*p* < 0.01).

**Table 6:** Comparison of shoulder tilt angle before and after intervention in each group.

Group	Left shoulder high			Right shoulder high		
	Before intervention	After intervention	*P*	Before intervention	After intervention	*P*
Football team	2.47 ± 2.45	1.47 ± 0.79	0.005**	0.23 ± 0.18	0.91 ± 0.61	<0.001**
Badminton team	1.92 ± 0.99	1.20 ± 0.81	0.112	0.65 ± 0.86	0.69 ± 0.31	0.959
Dance team	1.89 ± 1.09	0.94 ± 0.71	<0.001**	0.47 ± 0.08	1.00 ± 0.79	0.004**

**P < 0.01.

From Table 7, it can be seen that all three types of exercise increased the forward tilt angle of the pelvic sagittal plane, but significantly decreased the left tilt angle of the coronal plane (*p* < 0.01). They also improved the right rotation angle of the horizontal plane, with the most significant effect being observed in soccer (*p* < 0.01).

**Table 7:** Comparison of pelvic tilt angle before and after intervention in each group.

Group	Pelvis Sagittal Plane Forward tilt angle			Coronal plane of the pelvis Left tilt angle			Pelvic horizontal plane Right rotation angle		
	Before intervention	After intervention	*P*	Before intervention	After intervention	*P*	Before intervention	After intervention	*P*
Football team	7.37 ± 3.28	8.73 ± 2.99	0.003**	2.45 ± 1.76	1.41 ± 0.77	<0.001**	18.85 ± 20.00	6.85 ± 22.21	<0.001**
Badminton team	8.05 ± 3.33	9.18 ± 2.94	0.345	3.38 ± 0.91	1.39 ± 0.60	<0.001**	14.06 ± 3.50	21.96 ± 44.26	0.260
Dance team	7.00 ± 3.72	10.33 ± 6.60	<0.001**	2.43 ± 0.84	1.43 ± 1.09	<0.001**	14.70 ± 8.21	13.90 ± 38.80	0.881

**P < 0.01.

## 4 Discussion

This study investigated students aged 6–18 from primary school, junior high school to high school. The computer vision-based intelligent spinal testing system has no impact on students’ health and does not require highly specialized operator skills. Its operation is simple and convenient, making it suitable for large-scale spinal screening. However, certain variability may occur during testing. Two methods can be employed to minimize this variability: First, conduct accuracy validation against a gold standard to ensure the instrument operates within an acceptable margin of error. Second, perform three tests for each subject and use the average value as the final measurement result.

From the research results, the problem of neck forward tilt is significant among adolescents aged 6 to 18, with a high-risk proportion of 34.2%. The problem is relatively serious among low-age and 18-year-olds, and boys have a larger tilt angle than girls; A significant proportion of individuals exhibit a leftward tilt in their neck, predominantly on the right side. The rightward tilt angle is notably greater in males than in females, particularly among younger individuals and those aged 18; The proportion of high-risk individuals in the coronal neck angle was 3.3%, with a higher number of high-risk individuals at the ages of 6, 7, and 15. There was no significant difference between boys and girls. A study found that the abnormality rate of the sagittal and coronal planes of the neck was higher in boys than in girls (1). This study verified that the inclination angle of boys was also greater than that of girls. The forward neck posture problem among high school students is consistent with Yang Xin’s test results, with head-forward posture being one of the primary issues in spinal health and poor body posture among high school students (4). Pan (5) found in their research that the incidence of forward neck posture among college students was lower than the results tested in this study. This suggests that a significant portion of the forward neck posture problem in high school students may be attributed to prolonged study time and insufficient physical activity.

The number of adolescents aged 6–18 years with high-risk chest sagittal plane is relatively small, accounting for 0.2%. The high-risk group is mainly composed of 17- and 18-year-olds, with a tendency towards chest forward tilt; The number of people leaning to the right is slightly higher than that of leaning to the left, with 1.2% in the medium-high risk state. The number of people leaning to the left is higher in the high age group, and the angle of inclination is greater for girls than boys. The number of people leaning to the right is higher in the low age group, and there is no significant difference between boys and girls; The proportion of high-risk individuals in the coronal chest angle is 28.1%, and it is more severe in children aged 6–10 years old. There is no significant difference between boys and girls.

The waist is mainly inclined backward, with the largest inclination angle at 8 years old. 1% of adolescents are at high-to-medium risk, and all are 17- and 18-year-olds. The inclination angle of girls is significantly greater than that of boys; The coronal plane of the waist mainly tilts to the right, and the angle of inclination is smaller as the age increases. 0.6% of children and adolescents are at a medium-high risk, with no significant difference between boys and girls; The proportion of individuals at high risk in the coronal waist angle is 0.9%, all of whom are in the low age group and 18 years old. There is no significant difference between boys and girls.

The overall coronal plane of the spine is mainly tilted to the right, with a high-risk proportion of 9.6%. From the perspective of age, high-risk proportions are higher in the low- and high-age groups, with a relatively low proportion of high-risk in the 10–14 age group, and a lower inclination angle. From the perspective of gender, the angle of inclination of female students to the right is significantly greater than that of male students.

Only 0.1% of adolescents have no shoulder tilt, 96% of adolescents have a left shoulder that is higher than the right, and 15.3% of adolescents are at a medium-high risk. The proportion of adolescents at medium-high risk is higher among younger and 18-year-olds, and the tilt angle is larger. From the perspective of gender, the left shoulder height of boys is significantly higher than that of girls, which is consistent with the research of Liu et al. (6, 7).

The number of people with a forward tilt of the pelvic sagittal plane is 100%. The maximum tilt angle is at the age of 18, and 0.2% of children and adolescents are at a medium to high risk. The tilt angle of girls is significantly higher than that of boys, and girls are more likely to have pelvic forward tilt problems (6); The coronal plane of the pelvis is mainly tilted to the left, with a larger average tilt angle for ages 18 and younger, with no significant difference between boys and girls; The proportion of people with a medium-high risk of pelvic level is 29.6%, mainly due to right-handedness, with no significant difference between boys and girls.

**Figure 12:**
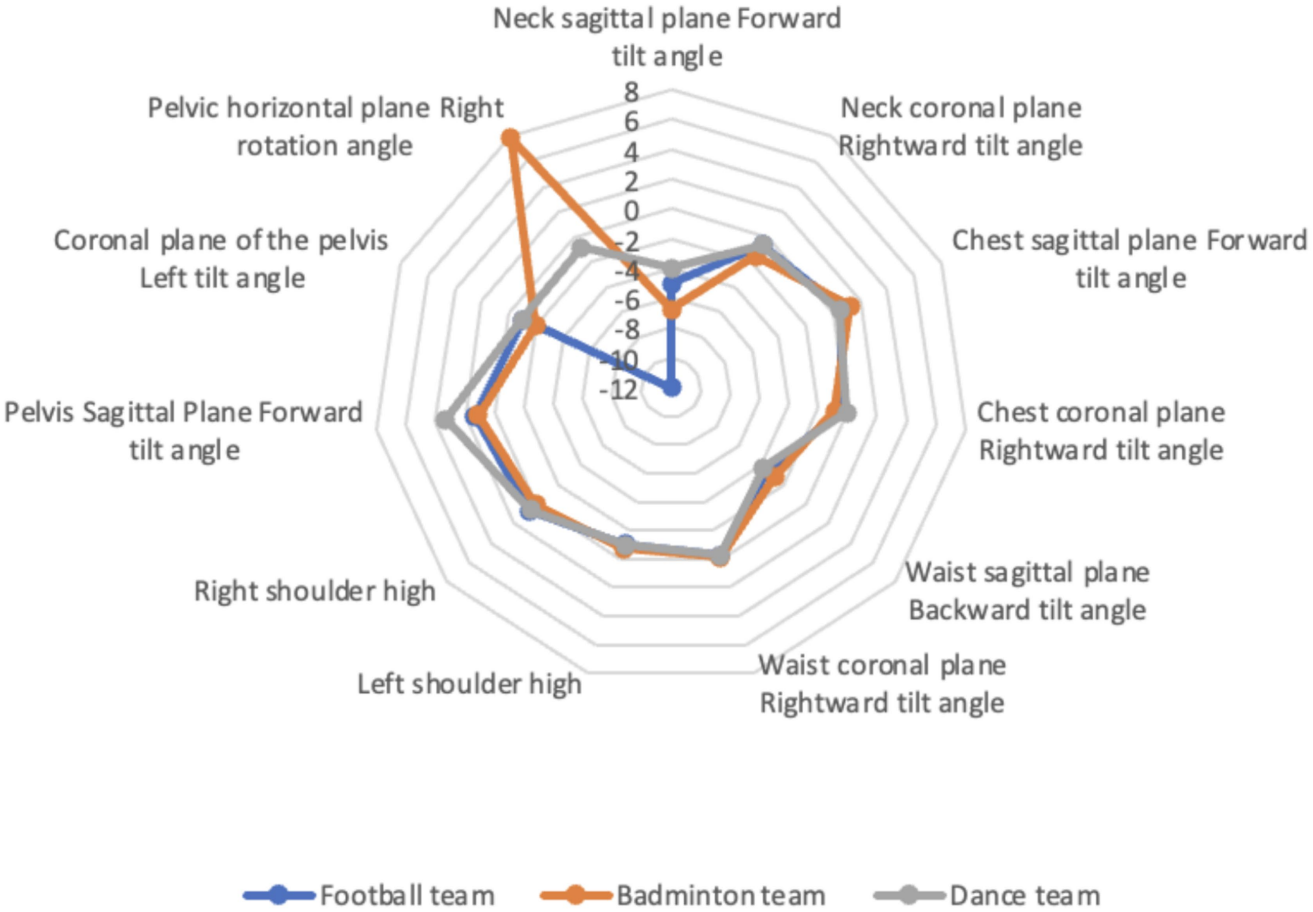
The effects of each exercise group on improving different spinal segments.

The research results are similar to previous studies, with more than 50% of adolescents exhibiting varying degrees of abnormal body posture (8), and a high incidence of abnormal neck forward tilt and high and low shoulder posture in adolescents (9). Previous studies have shown that the abnormality of adolescent body posture increases with age (10). The results of this study show that spinal problems are more serious in the lower age groups and 18-year-olds. In some spinal segments, problems are more serious in the graduating grades of primary, junior high, and high school. The difference in results may be due to the fact that previous studies only investigated adolescents in primary or middle school, while this study investigated the entire primary and secondary school stage from 6 to 18 years old, which is more accurate. Spinal issues in younger children may occur due to softer bones, where short-term poor postures can lead to temporary spinal curvature abnormalities. However, as their bones and soft tissues are in a phase of rapid growth and high plasticity, these issues can be quickly corrected with proper intervention and guidance. In contrast, 18-year-old adolescents may develop spinal health problems due to factors such as prolonged sitting, sustained poor postures, and psychological stress associated with the pressure of college entrance exams. The reasons for the gender differences remain unclear, and future research could explore the underlying mechanisms based on these differences (Figure 12).

According to research and literature, sedentary behavior, insufficient physical activity, incorrect sitting posture, excessive screen time, and sleep and diet are important factors that affect the spine health and body posture of adolescents (9–11). Therefore, good living, learning and exercise habits are beneficial to promoting the healthy development of adolescents’ body and spine.

Previous studies have mostly focused on the impact of certain forms of exercise on body posture (12–16). This study compared the effects of lower-extremity-dominant soccer, upper-extremity-dominant badminton, and full-body symmetrical exercise such as aerobics on body posture. The experiment conducted a 12-week intervention of soccer, badminton, and aerobic dance on 169 subjects. Except for the forward tilt of the chest sagittal plane and the right shoulder height, the three forms of exercise improved the tilt angles of different directions in other parts to varying degrees. Following the intervention, both the football and badminton groups showed a significant increase in thoracic inclination angle. This indicates that the movements involved in football and badminton may be detrimental to the health of the thoracic spine. The dance group also exhibited a slight increase in the inclination angle, which is likely related to dance movements that frequently involve a rounded shoulder posture, potentially leading to an increased sagittal plane inclination of the thorax. Therefore, when designing dance routines, it is important to incorporate more chest-expanding movements to improve the inclination angle of the thorax in the sagittal plane. Although all three types of exercise significantly improved the angle of lumbar tilt, the angle reduced the most in the aerobic dance category. The waist coronal plane rightward tilt angle also showed no significant difference before and after the intervention (*p* > 0.05). The possible explanation is that while adolescents’ initially excessive lumbar curvature in the sagittal plane (likely caused by poor sitting posture and prolonged studying or mobile phone use with lowered head) could be significantly improved through physical activities, the pre-existing lateral abnormal curvature in the coronal plane was relatively minor to begin with, making the intervention’s effect on this parameter less noticeable. All three exercises improved the left shoulder height, which is consistent with other studies (17, 18). However, they all improved the right shoulder height, which may be due to insufficient sample size for the right shoulder height. During the screening assessment process, it was found that 96% of the subjects had left shoulder height. Badminton has improved the left shoulder height, but the improvement is smaller than that of football and dance sports, which may be related to the asymmetric movements of badminton. The three exercises did not improve pelvic tilt, which is inconsistent with some studies. This may be due to age differences, as it was found in the survey that the problem of pelvic tilt was most severe among 18-year-olds. The three sports have a greater improvement in the left tilt angle of the pelvic coronal plane, with soccer having the most significant improvement in the right rotation of the pelvis. Therefore, whether it is football, badminton or aerobics, the benefits of correcting the spine outweigh the disadvantages, and aerobics have a greater effect on improving the tilt of the waist. Football has a greater effect on improving the right rotation of the pelvis, while badminton is not suitable for improving the height of the shoulders.

## 5 Conclusion

The results of the survey and testing of the sagittal and coronal planes of the neck, chest, and waist, the overall coronal plane of the spine, the sagittal and coronal planes of the shoulder, and the sagittal, coronal, and horizontal planes of the pelvis of 6–18-year-old adolescents showed problems, with the neck, shoulder, and pelvis being more severe.

There are age and gender differences in the spine health of adolescents aged 26–18, with problems being more prominent in the lower age groups and among those aged 18.

Football, badminton, and aerobics have more advantages than disadvantages for correcting the spine, and aerobics have a greater effect on improving the tilt of the waist. Football has a greater effect on improving the right rotation of the pelvis, while badminton is not suitable for improving the height of the shoulders. Therefore, when using exercise to improve spinal health issues, comprehensive exercises can be employed to holistically address poor spinal conditions. However, problems in different spinal segments require targeted movements to correct the inclination angles of specific areas. For example, for adolescents with severe thoracic kyphosis, we can incorporate specific movements like chest expansion exercises solely to address the issue of excessive thoracic kyphosis. Regardless of the type of exercise intervention, the intensity and frequency should be adjusted according to individual circumstances.

In future research, non-exercise control groups and knowledge education groups could be added to fully illustrate the impact of various exercises on spinal health. Additionally, incorporating other factors influencing spinal health such as participants’ physical activity, lifestyle habits, and sleep patterns would enable a comprehensive exploration of the factors affecting adolescents’ spinal health.

## Data Availability

The raw data supporting the conclusions of this article will be made available by the authors, without undue reservation.
